# Prefrontal cortex connectivity during right and left hand dexterity tests in younger and older adults

**DOI:** 10.1371/journal.pone.0342547

**Published:** 2026-02-12

**Authors:** Manjiri Kulkarni, Peter Rassam, Pedram Mouseli, Monisha Date, Tamires Mori, Marine Van Hollebeke, Jennifer L. Campos, Dmitry Rozenberg, W. Darlene Reid

**Affiliations:** 1 Rehabilitation Sciences Institute, University of Toronto, Toronto, Ontario, Canada; 2 Department of Physical Therapy, University of Toronto, Toronto, Ontario, Canada; 3 Toronto General Hospital Research Institute, University Health Network, Toronto, Ontario, Canada; 4 Centre for Multimodal Sensorimotor and Pain Research, Faculty of Dentistry, University of Toronto, Toronto, Ontario, Canada; 5 University of Toronto Centre for the Study of Pain, Toronto, Ontario, Canada; 6 KU Leuven, Department of Rehabilitation Sciences – Leuven, Belgium; 7 KITE - Toronto Rehabilitation Institute, University Health Network, Toronto, Ontario, Canada; 8 Division of Respirology, Temerty Faculty of Medicine, University of Toronto, Toronto, Ontario, Canada; 9 West Park Health Care Centre, University Health Network, Toronto, Ontario, Canada; 10 Interdepartmental Division of Critical Care Medicine, University of Toronto, Toronto, Ontario, Canada; Nathan S Kline Institute, UNITED STATES OF AMERICA

## Abstract

**Background:**

Age can negatively impact activation and connectivity among prefrontal cortex (PFC) regions during demanding motor functions, such as hand dexterity tasks. The study objective was to compare functional connectivity between PFC regions during right and left-hand 9-hole peg tests (R9HPT and L9HPT) in younger versus older adults.

**Methods:**

Two groups were tested: 20 Younger (29 ± 4 years; 10F:10M) and 19 Older (67 ± 12 years; 10F:9M) adults. Healthy right-handed adults performed timed R9HPT and L9HPT. Functional near infrared spectroscopy (fNIRS), positioned over eight PFC regions, provided a surrogate measure of neural activity from baseline to test completion as the change in oxygenated hemoglobin (∆O_2_Hb). Time series analysis of ∆O_2_Hb evaluated connectivity between pairs of PFC regions.

**Results:**

R9HPT and L9HPT test completion times were longer for Older than Younger adults (*p* ≤ 0.001) and secondly, longer during L9HPT than R9HPT for both groups (p ≤ 0.001). Greater ∆O_2_Hb was associated with longer test completion times in 5 of 8 PFC regions in Older adults. Patterns of functional connectivity differed between R9HPT and L9HPT within group and between groups (Younger and Older); both groups showed connectivity between the right lower dorsolateral and left upper medial regions during the L9HPT. The left upper medial, right upper medial, left lower medial and left dorsal lateral were mostly involved with other PFC regions. Interhemispheric PFC connectivity was demonstrated for R9HPT and L9HPT for both age groups. Significant intrahemispheric PFC connectivity was shown for R9HPT and L9HPT in the Older group but only in L9HPT for the Younger group.

**Conclusion:**

Functional connectivity of the PFC evaluated by fNIRS not only differed in Older versus Younger adults but the ∆O_2_Hb, a surrogate of neural activity, showed more pairing between regions during hand dexterity by the non-dominant hand. Age and non-dominant hand required more connectivity but did not lead to improved task efficiency.

## Introduction

Manual dexterity facilitating skillful hand function is essential for various self-care tasks and activities of daily living (ADL) [1]. Decline in manual dexterity with age could lead to loss of independence. Age-related changes can negatively affect the neural systems required for manual dexterity, such as tactile perception, sensorimotor (vision and motor) functions, co-ordination, and cognitive processing speed [2–4]. This decline can be due to the age-related changes in the cortical activation patterns, including over-activation, evidenced to be more prominent from 50 years of age [5]. One cortical region of interest is the prefrontal cortex (PFC), which regulates motor control and the cognitive processes required for manual dexterity tasks [6]. Several medical conditions, which increase in prevalence with older age such as cardiopulmonary diseases (i.e., heart disease, chronic obstructive pulmonary disease) or neurological conditions (i.e., stroke), can also contribute to decrements in dexterity [7].

Previous studies using neuroimaging techniques have demonstrated that compared to younger adults, older adults exhibit an increase in PFC activation during motor tasks, along with less lateralization and greater bilateral activation of this region. Some of these studies have interpreted this age-related difference as compensation and others as maladaptive dedifferentiated neural activity. Neural compensation strategies may be used when a task is relatively complex, needing more cortical areas to perform the task efficiently. In contrast, maladaptive dedifferentiated strategies assert that decrease in inhibition of interhemispheric connection among regions, requiring greater bilateral involvement may contribute to performance decline, especially in older adults [8]. Examining functional connectivity patterns across age groups within PFC regions using functional near infrared spectroscopy (fNIRS) may help to understand which of these two strategies may be evident during manual dexterity tasks.

Brain activity during physical tasks that require more movement can be evaluated by continuous wave fNIRS, with the change in oxygenated hemoglobin (ΔO_2_Hb) as the marker of neural activity. By placing the fNIRS optodes on the forehead, the neural activity of medial and dorsolateral PFC regions bilaterally can be estimated [10,11]. It can also provide information regarding the inter- and intrahemispheric functional connectivity among regions. The term functional connectivity refers to the connections between brain regions. Mapping these connections during task performance across different age groups can show the age-related differences in regional connectivity [12]. Another consideration for task performance is that hand dominance can play an important role in performance and connectivity, as right-hand dominant individuals may perform manual dexterity tasks more automatically with their right hand and require fewer neural resources with less connectivity evident. The 9-hole peg test (9HPT), which assesses manual dexterity, is considered the gold standard endorsed by the National Institutes of Health (NIH) [13], thus was chosen for the current study to evaluate manual dexterity.

Functional connectivity has been assessed using functional magnetic resonance imaging (fMRI) during the performance of various manual dexterity tests (i.e., finger tapping, fist clenching, thumb to index finger) [8,9]. While fMRI has restrictions (supine and inability to move), using fNIRS in the current study enabled monitoring of neural activity during a more complex task in an upright position that requires visualization and movement of the trunk and upper extremity joints, including the hand. This evaluation mirrors the complexity of daily manual dexterity requirements with ADL. Building on this rationale, the objective of this study was to compare functional connectivity among PFC regions while performing a right-handed 9-hole peg test (R9HPT) and a left-hand 9-hole peg test (L9HPT) and its association with test completion times in Younger versus Older adults. The Older group was postulated to demonstrate greater functional connectivity among PFC regions during both tasks, particularly with the non-dominant hand. Further, it was hypothesized that longer test completion time would be associated with greater changes in neural activity and more connectivity among regions.

## Materials and methods

### Participant characteristics

Participants were recruited from the University of Toronto campus and the surrounding community through emails and flyers. Inclusion criteria were: (a) healthy adults of both sexes within 18–40 years of age for the younger group, and ≥ 50 years for the older group; (b) non-smokers (or ex-smokers of less than 10 pack/years) due to the potential widespread differential systemic effects of smoking; it has been shown to alter manual dexterity and vascularity affecting fNIRS data [14]; (c) sufficient English fluency for understanding the consent form and following study instructions; (d) right-hand dominant. Right-hand dominance was confirmed with the Edinburgh Handedness Inventory (EHI) [15]. Exclusion criteria were: (a) acute or chronic diseases of respiratory, cardiovascular, musculoskeletal, or neurological systems that could potentially affect the test performance referring to the American College of Sports Medicine (ACSM) questionnaire [16]; (b) self-reported poor vision or hearing (even with hearing and visual aids) from the Canadian Longitudinal Study of Aging (CLSA) questionnaire [17,18].

### Experimental protocol

**Fig 1:**
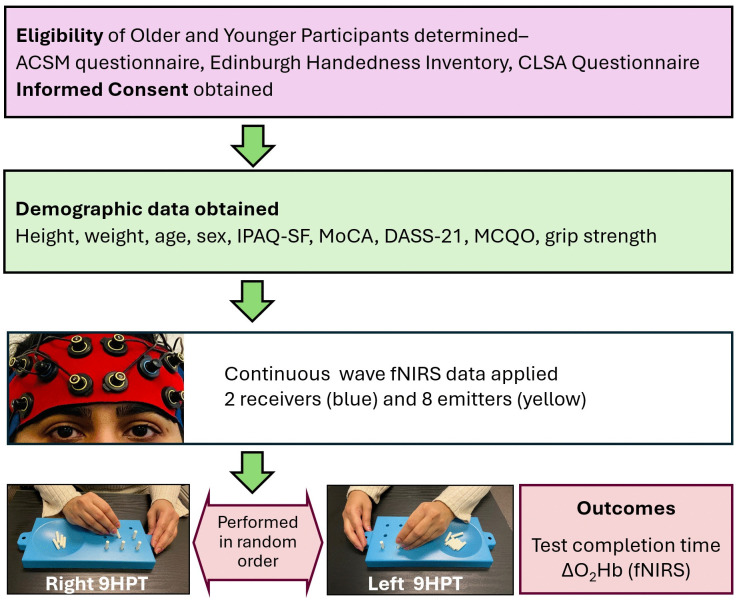
Study Design Overview. Experimental flow of eligibility, collection of demographic data including questionnaires, fNIRS application and performance of the right and left 9-hole peg tests in random order. *ACSM = American College of Sports Medicine; CLSA = Canadian Longitudinal Study of Aging; IPAQ-SF = International Physical Activity Questionnaire-short form; MoCA = Montreal Cognitive Assessment, DASS-21 = Depression, Anxiety, Stress score-21, MCQO = Medication and Comorbidities Questionnaire, fNIRS = Functional near-infrared spectroscopy, R9HPT = Right hand 9-hole peg test; L9HPT = Left hand 9-hole peg test.*


This study was a single-visit cross-sectional design (**Fig 1**), approved by the University of Toronto Research Ethics Board (#43849). Ethics was initially approved Jan 1^st^, 2023, and was renewed on a yearly basis subsequently. Participants were recruited from 19^th^ May 2023 until 5^th^ December 2023. Written informed consent was obtained from each participant prior to study enrollment. A thorough explanation of the study processes and opportunity to ask questions was provided as per research ethics guidelines. Age, sex, weight, height and body mass index were recorded. Participants completed questionnaires that queried cognitive status, comorbidities and physical activity. After grip strength was evaluated, the fNIRS sensor headband (**Fig 1**) was fitted over participants’ foreheads to monitor the ΔO_2_Hb in the PFC regions. Participants performed the 9HPT for the right- and left-hand (R9HPT and L9HPT) in a randomized and counterbalanced fashion, while the test completion time and fNIRS data were recorded. Mean blood pressure was measured on the right arm, whereas heart rate and oxygen saturation were assessed on the left-hand index finger at rest and immediately after the R9HPT and L9HPT (**Fig 1**). Mean arterial pressure (MAP) was calculated with the formula -

MAP = Diastolic Blood Pressure + 1/3 * (Systolic Blood Pressure − Diastolic Blood Pressure).

Functional near-infrared spectroscopy (fNIRS): The PFC ΔO_2_Hb was obtained using a continuous-wave NIRS-system (OctaMon, Artinis, The Netherlands) through application of an eight-channel sparse grid array designed for acquisition over the PFC [19]. The system consists of two receivers and 8 emitters of infrared light at two wavelengths, 730nm and 850nm to detect deoxygenated and oxygenated hemoglobin, respectively. The interoptode distance was 30 mm. The light intensity raw data were sampled at 10 Hz by Oxysoft software (Aritnis, The Netherlands) [20,21]. S1 Fig shows the location and associated nomenclature that describes the channels. One of two sizes of bands were fitted to the participants that were either 20 or 25 mm between the medial channels (S1 Fig). *For the fNIRS connectivity analyses, fNIRS signals (ΔO*_*2*_*Hb) were bandpass filtered (cut off frequency: 0.01 to 0.2; filter type: Butterworth order 3)*

9-hole peg test (9HPT): The Jamar 9-hole peg test was used, which is comprised of a moulded dish next to the 9-hole peg (31 × 26 × 4 cm) with 9 plastic pegs (0.6 cm in diameter). The table height was adjusted so that the forearms rested comfortably on the tabletop at mid-waist level. Using standardized instructions, participants were instructed to place the pegs into the pegboard using one hand followed by removing the pegs and placing them in the dish [23]. The test completion time (seconds) was determined for the R9HPT and L9HPT separately [13]. The test was demonstrated prior to testing; time to pick-up a dropped peg was incorporated into the test completion time. Its applicability across age groups, psychometric soundness, short completion time (brevity), applicability in diverse settings make it an ideal test across age groups. 9HPT has a test-retest reliability of 0.95 and 0.92 for the right and left hand, respectively [13,22,23]. The normative values are available for 9HPT across various age groups [22,23] and existing studies support its reliability and validity for use with various age groups [13,22,23].

### Questionnaires and baseline assessments to characterize participants

Participants completed questionnaires and baseline assessments before performance of the 9HPTs.

#### Montreal Cognitive Assessment (MoCA).

This 30-point measure of cognition provides a total score based on sub scores in the following domains: attention, short-term memory, concentration, and executive function [24]. Education level was ascertained as part of this questionnaire.

#### Depression, Anxiety, Stress Scale 21 (DASS-21).

The modified 21-item scale was used to assess feelings of stress, anxiety, or depression that participants may have experienced during the last 7 days ascertained on a scale from 0 to 3, with 0 being nothing at all and 3 being maximum [25].

#### Medication and Comorbidities Questionnaire (MCQO).

This questionnaire provides an indication of self-reported number of comorbidities and medications for each participant [26,27].

#### International Physical Activity Questionnaire – Short Form (IPAQ-SF).

This 7-point questionnaire characterizes the physical activity levels over a one-week period reflected by total physical activity in Metabolic equivalent (MET)-min/week [28].

#### Grip strength.

The maximum voluntary contraction (MVC) of handgrip force was evaluated for the right hand using the grip force transducer (MLT004/ST, ADInstruments, Colorado Springs, CO, USA) connected to LabChart/PowerLab (ADInstruments). Only the right hand grip strength was recorded as it was done as a characteristic measure and not as a predictor or outcome variable. While providing standardized encouragement, participants completed three assessments with two-minute rests between contractions. The highest force produced was recorded as the MVC [29].

### Statistical analysis

An effect size of 0.906 was calculated from 9HPT normative values of test completion time for age groups of 26–30 years and 56–60 years [13], using G*Power software (version 3.1.9.7). Application of effect size 0.906, an alpha error probability of 0.05 and power of 0.80 provided a sample size estimate of n = 16 per group to observe a difference in 9HPT between the two age groups. Samples of 20 per group were targeted to account for potential drop-outs and missing data.

Statistical analyses were conducted using IBM SPSS (version 29) and Python (3.12). Python was used for the connectivity analysis of fNIRS data*.* After testing for normality by the Shapiro-Wilk test, descriptive statistics were performed to summarize the sample (means, SD, medians and interquartile ranges) within each group. Group differences between 9HPT test completion times, participant characteristics and baseline assessments, and physiological outcomes were tested using unpaired t-tests or Mann-Whitney U tests depending on normality and nature (ordinal, continuous) of the data. Examination of correlations of the maximal ΔO_2_Hb between regions and the correlations of the maximal ΔO_2_Hb in a specific PFC region with age were performed using Pearson Correlation. Multiple comparisons were corrected for using modified Bonferroni [30].

For the fNIRS connectivity analyses, fNIRS signals (ΔO_2_Hb) were filtered and Pearson correlation coefficients were computed between the time series of all 8 regions within each subject and each task (R9HPT, L9HPT) to examine functional connectivity. Test completion times varied between tasks and among participants, but the entire duration for each task was normalized prior to Pearson correlation. This resulted in a symmetric 8x8 connectivity matrix per participant and test. The raw correlation values were then transformed using the Fisher’s r-to-z method. Next, each connectivity matrix was z-scored (normalized by its mean and standard deviation). One-sample t-tests were performed within each test (R9HPT, L9HPT) and age group (Younger, Older) for each connectivity (null hypothesis: connectivity mean = 0). P-values were corrected for multiple comparisons using False Discovery Rate (FDR) within each test and group. Statistically significant values (FDR-corrected *p* < 0.05) were visualized to identify functional connectivity between pairs of PFC regions [31].

## Results

A total of 39 right-hand dominant participants were recruited, with 20 in the Younger group and 19 in the Older group, and approximately equal sex distribution for both groups. The Younger group had a mean age of 29 ± 4 years and the Older group had a mean age of 67 ±12 years (p < 0.001). There were no significant differences in participant characteristics between the two groups, aside from age and greater number of comorbidities in the Older group, as shown in **Table 1**. All participants had a minimum of a high school education.

**Table 1:** Characteristics of participants in Younger and Older groups.

Participant Characteristic	Younger	Older	*p* value
Age (Years)	29 ± 4	67 ±12	**<.001**
Sex (n of Females: Males)	10:10	10:9	.869
Body Mass Index (kg/m^2^)	23.2 ± 2.2	23.3 ± 2.6	.898
DASS-21	11 [2, 21]	6 [1, 14]	.180
Handgrip (Newton)	344 [268, 430]	282 [216, 359]	.089
Montreal Cognitive Assessment	28 [26, 29]	29 [27, 29]	.675
Number of Comorbidities	0 [0,0]	1 [0, 3]	**.008**
Number of Medications	0 [0,0]	0 [0,2]	.061
IPAQ – Activity Level (MET-min/week)	3937 [2932, 7616]	3626 [2243, 7182]	.279

Data is provided as mean ± SD or as median [interquartile range]. *DASS-21 = Depression, anxiety, stress scale, IPAQ = International Physical Activity Questionnaire.*

**Fig 2:**
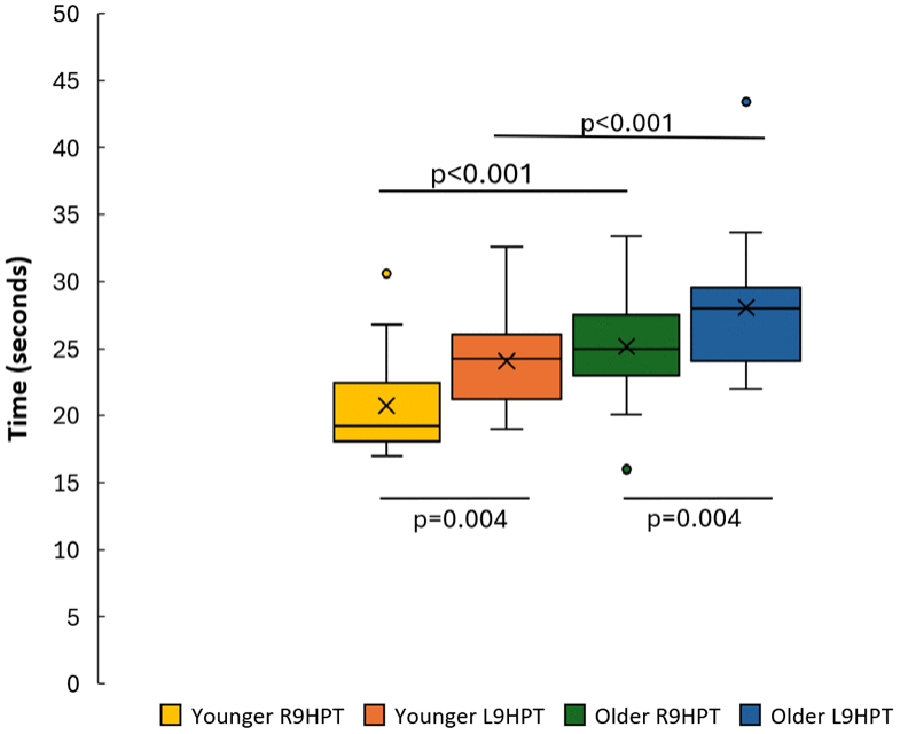
Test completion times by group and by task. p < 0.001 represents the significance between groups and p = 0.004 represents the significance within groups. The median is the midline of the box with the upper edge showing the 75^th^ percentile and the lower edge showing the 25^th^ percentile. The whiskers show the range except if outliers are present (greater than 1.5 times the interquartile range) which are represented by the dots. Means are shown with x. *R9HPT = Right hand 9-hole peg test; L9HPT = Left hand 9-hole peg test.*

Comparisons between groups showed that the test completion times were significantly shorter for the Younger group than those of the Older group for the R9HPT and L9HPT (p < .001; **Fig 2**). Younger group was 21% faster during R9HPT and 17% faster during L9HPT than the Older group. Comparisons between sides showed that R9HPT durations were significantly shorter than L9HPT for both the Younger and Older groups (p = .004; **Fig 2**). Comparing between sides, R9HPT was 16% and 12% faster than the L9HPT in the Younger and Older group, respectively. Among the Older group, 2 participants dropped the pegs during L9HPT while directing them into the holes and the time for pick-up and continuation of the test was reflected in the test completion time.

### fNIRS time series connectivity

**Fig 3:**
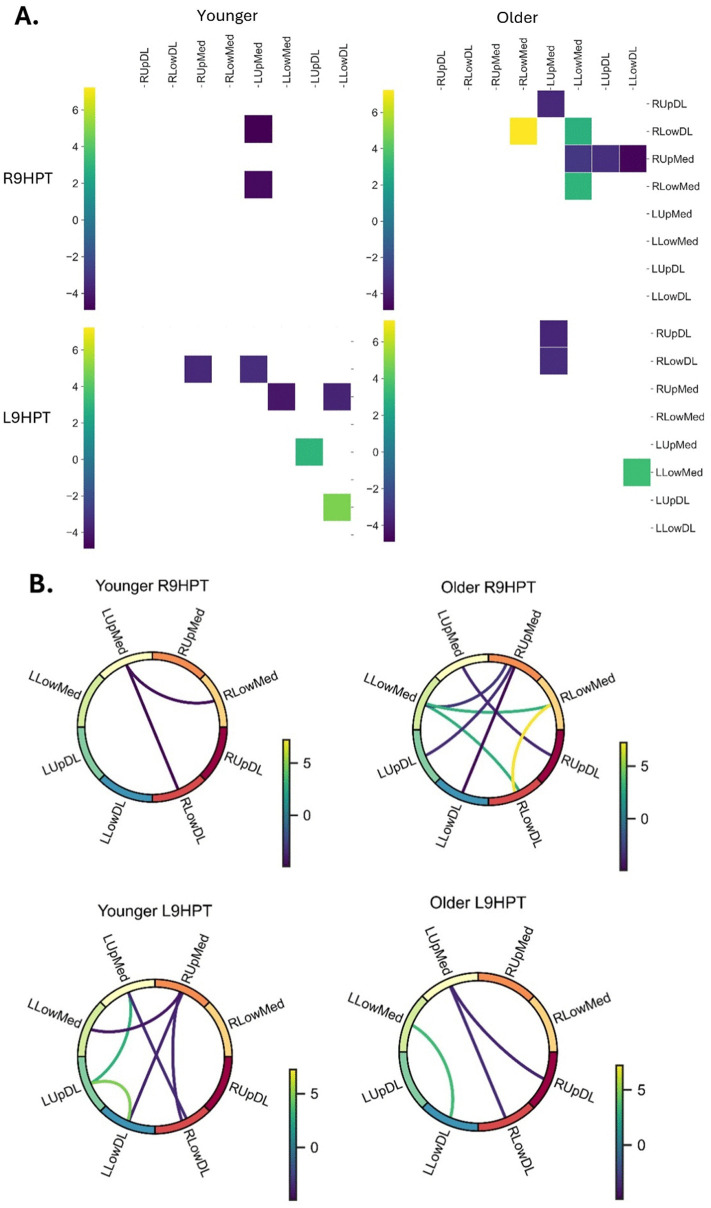
Time series correlations between 8 prefrontal cortex (PFC) regions within groups and two 9HPTs and corresponding directional functional connectivity between pairs of PFC regions. ***Panel A*** shows the significant t statistic values of the time series correlation among 8 regions of the prefrontal cortex within a group (Younger and Older) and by test (R9HPT and L9HPT). The colour of the vertical legends depicts the t-statistic score values. Only significant pairs are shown, corrected with false discovery rate method for multiple comparisons. **Panel B** provides a diagrammatic representation of functional connectivity between the channels of PFC regions by group and task. *RUpDL = Right Upper Dorsolateral; RLowDL = Right Lower Dorsolateral; RUpMed = Right Upper Medial; RLowMed = Right Lower Medial; LUpMed = Left Upper Medial; LLowMed = Left Lower Medial; LUpDL = Left Upper Dorsolateral; LLowDL = Left Lower Dorsolateral; R9HPT = Right hand 9-hole peg test; L9HPT = Left hand 9-hole peg test.*

During the R9HPT, the Younger adults demonstrated less functional connectivity (fewer significant t-tests) between pairs of PFC regions as compared to the Older adults (**Fig 3A**). However, during L9HPT, Younger adults showed more functional connectivity between pairs of PFC regions than the Older group. **Fig 3A** provides significant effects, whereas **Fig 3B** shows the functional connectivity between significantly associated pairs of PFC regions. With respect to similarity between groups of significant related pairs of PFC regions, both Younger and Older groups during L9HPT demonstrated connectivity between the right lower dorsolateral (RLowDL) and the left upper medial (LUpMed) regions. Across all tasks, the most involved regions in functional connectivity were the left upper medial (LUpMed), followed by the right upper medial (RUpMed), left lower medial (LLowMed) and left lower dorsolateral (LLowDL). Both groups demonstrated interhemispheric PFC connectivity during both right and left 9HPT tasks. However, only the Older group showed intrahemispheric PFC connectivity for both tasks, while the Younger group exhibited it only during the L9HPT.

### *Age group with Change in O*_*2*_ *Hb*

Increasing age was associated with a greater ΔO_2_Hb during the L9HPT in the Older group in 5 out of 8 PFC regions: right upper dorsolateral (RUpDL), right lower dorsolateral (RLowDL), right lower medial (RLowMed), left lower medial (LLowMed) and left lower dorsolateral (LLowDL) (see S1 Table). No other correlations of age with ΔO_2_Hb were shown during the tests except for ΔO_2_Hb in the RLowMed for the R9HPT for the Younger group.

*Correlations of the magnitude of change in O*_*2*_*Hb* (ΔO_2_Hb) by group (Younger and Older) and by task (R9HPT and L9HPT) among 8 regions are provided in S2 Fig. The number of significant correlations from a potential total of 28 unique pairs of PFC regions was similar for R9HPT and L9HPT in the Older group (12 out of 28 for both). In contrast, the Younger group showed that ΔO_2_Hb was significantly correlated in the 11 of 28 pairs and all of the 28 potential pairs of PFC regions for the R9HPT and L9HPT, respectively (S2 Fig).

Cardiorespiratory status (heart rate (HR), mean arterial pressure (MAP) oxygen saturation (SpO_2_) were not different between groups at baseline and after tests except the Older group had a higher MAP immediately after the R9HPT (p = 0.032) compared to the Younger group (see S2 Table).

## Discussion

This study aimed to compare the functional connectivity among PFC regions during right versus left 9HPT between Younger and Older adults, along with test completion times, to explore if altered connectivity with increasing age impacts manual dexterity based on hand dominance. The Younger group exhibited more paired connectivity among PFC regions within and across PFC hemispheres (right and left) during L9HPT, while the Older group showed more paired connectivity among PFC regions within and across PFC hemispheres during R9HPT. However, the Older group during L9HPT demonstrated lesser paired connectivity within and across PFC hemispheres, with the Younger showing the least paired connectivity among PFC regions, limited only to interhemispheric connections during dominant right hand 9HPT. Longer 9HPT test completion times did not correspond with greater functional connectivity among the PFC regions or a greater ΔO_2_Hb. However, older age was associated with a greater ∆O_2_Hb during L9HPT. These differences in connectivity and ∆O_2_Hb were derived during the expected longer completions times of the non-dominant versus dominant 9HPT and in Older compared to Younger participants.

Functional connectivity, expressed as a statistical dependence between time series, can indicate how the connectivity changes between PFC regions throughout a task [12]. Upon diagrammatic representation of functional connectivity among the pairs of PFC regions, the Younger group during R9HPT demonstrated fewer interhemispheric regions of connectivity, which may be attributed to easier, automatic motor control due to its more frequent use during ADL in right hand dominant adults. The same group while performing the L9HPT, evidenced interhemispheric and a few intrahemispheric connections while maintaining task efficiency and greater lateralization. A greater number of channels showed functional connectivity during L9HPT, which can be considered in alignment with the adaptive compensation strategy, as the task with the non-dominant hand can be perceived as more complex and requiring the connectivity of more PFC regions for task completion [8]. Even though within group L9HPT was slower, between groups showed that Younger L9HPT was quicker than Older L9HPT. However, the Older group showed more patterns of bilateral cortical activation with more inter- and intrahemispheric connections among pairs of PFC regions only observed during R9HPT.

In spite of the greater number of connections for the Older group during the R9HPT, completion times were longer than those of younger adults, which fits with the dedifferentiation strategy. In other words, even with greater diffuse connectivity in Older adults, their behavioural performance did not match that demonstrated by the Younger group. Based on the mixed findings from the literature, some studies have shown a greater increase in PFC activation in the older group during upper limb [32] tasks, whereas in some studies older individuals demonstrated smaller ∆O_2_Hb in this region [33]. There is also evidence that increased complexity of the task (L9HPT in this case) also leads to greater cortical activation in older adults than younger adults [34]. In the current study, greater ΔO_2_Hb was associated with older age for the L9HPT, however, there were not parallel increases in the number of connectivity patterns [8,35]. A probable explanation can be that L9HPT was a more complex task for right hand dominant older adults, and even though increases in activation were observed, it did not result in equal performance to that of the Younger group. Among all the tasks, L9HPT for the Older group was the slowest and resulted in less connectivity among the PFC regions.

Many of the PFC regions demonstrated an increase in ∆O_2_Hb during tasks in both groups, greater in the Older group with the complex task of L9HPT. This region has been known to show increased cortical activation patterns in older adults during motor task performance of hand-foot coordinated activities (both limb segments movement in the same and opposite directions) [8,36]. Voluntary visual attention and focus required to pick each of the pegs and place them in the correct order on the peg can also lead to PFC activation [37]. One of the instructions while performing this test was to do it as ‘quickly as possible’. This can also add an emotionally stressful component, and although not measured in this study, may have contributed to increased PFC activation [38]. Thus, the combination of the manual dexterity task, age, visual attention and the anticipatory perception of task completion can lead to an increased PFC activation. All together, functional connectivity of the PFC regions helps us understand the changes in the interactions among regions required for different tasks. This can aid with exploration of the neural activity necessary for various tasks such as grooming, managing medications or using an inhaler. Time series analysis with fNIRS data can be advantageous over other modalities such as fMRI in understanding the functional connectivity required for more complex manual dexterity tasks, which require performance in an upright position that could more closely simulate the demands of ADL.

Older adults took longer to complete both R9HPT and L9HPT compared to the Younger group. As previously shown, a decrease in motor and executive functions occurs with increasing age, which could manifest as longer test completion time [39]. Age related changes in hand dexterity have been less commonly studied but are estimated to reduce by about 10–30% with increasing age [40–43]. Further, handedness did have a limiting effect on function as the non-dominant hand took longer to complete the same task, as supported by the literature. L9HPT was slower within both groups, emphasizing that it took longer to complete this task using the non-dominant hand. One possible way of interpreting the findings could be with the hemi-aging theory, which attributes the effect of aging on cerebral dominance with a greater decline in the right dominant hemisphere that can lead to impairments in left-hand performance [44,45]. Slower L9HPT than R9HPT in the Younger group may be a result of having less practice with object manipulation and precision, requiring further investigation [44].

Decreases of sensorimotor and motor control functions are associated with a number of co-morbidities and chronic conditions that increase with age [46,47]. This can impact task execution resulting in reduced automatic movements. However, it is important to highlight that this study represents relatively healthy adults, with a low median number of comorbidities (median = 1). A lower age limit of ≥50 years was selected because several physiologic mechanisms that contribute to coordinated movement have been demonstrated to decline well before the chronologic age of 65 years including a loss of skeletal muscle fibres [48], motor control of hand dexterity as reflected by normative values [22,23] and connectivity of the prefrontal cortex [49–51]. In addition, the number of comorbidities increase exponentially between 40 and 50 years of age [52] and is noted by additional scoring on the Charlson Comorbidity index score for ages 50 years and older [47]. Future studies should explore more complex hand tasks that are representative of manual dexterity required in daily living and potentially evaluate participants with a higher burden of comorbidities to identify the impact on day-to-day activities.

### Limitations

The hand dexterity test was only assessed in right hand dominant adults, limiting the ability to observe any similarities or differences in neural activation that one may observe with cognitive-motor processing with hand dominance. Left hand dominant adults should also be tested to see the difference in PFC activity. The results from this study may not necessarily be translatable to individuals with significant comorbidities or cognitive impairment as majority of our participants had minimal comorbidities. This study also had a relatively lower age limit for Older adults, with individuals possibly highly functional at work and daily activities in their fifties and sixties, which may influence the contribution of cognitive abilities on hand dexterity tasks. Future work could investigate if fNIRS connectivity during hand dexterity tasks is differentiated among more specific age ranges of middle, older and elderly adults. Even though continuous wave fNIRS is a robust, portable neuroimaging modality, anatomical regional specificity is lower than other imaging tools such as fMRI.

## Conclusion

PFC neural connectivity differed between groups (Younger and Older) and tasks (right and left hand). The Older group showed more inter and intrahemispheric connections of neural activity amongst PFC regions, whereas the Younger group had fewer intrahemispheric connections, none of which were observed using the right hand. Older adults performed the hand dexterity test more slowly and both age groups had inferior performance with their non-dominant hand. Of clinical relevance, it would be worthwhile exploring the correlation between 9HPT and hand dexterity of daily activities and secondly, whether training is task specific or if there is a carry over across hand dexterity tasks.

## Supporting information

S1 Table Correlations between age and ΔO_2_Hb in 8 PFC regions. In the Older Group, older age was correlated with a larger ΔO_2_Hb in 5 out of 8 PFC regions during the L9HPT. Significant values are bolded. Abbreviations: *R9HPT: Right hand 9-hole peg test; L9HPT = Left hand 9-hole peg test; PFC = Prefrontal cortex; RUpDL = Right Upper Dorsolateral PFC; LUpDL = Left Upper Dorsolateral PFC; RLowDL = Right Lower Dorsolateral PFC; LLowDL = Left lower Dorsolateral PFC; RUpMed = Right Upper Medial PFC; LUpMed = Left Upper Medial PFC; RLowMed = Right Lower Medial PFC; LLowMed = Left Lower Medial PFC.*
(DOCX)

S2 Table Cardiovascular status measured at baseline and after each task. Data presented as median and interquartile range. Data presented as median and interquartile range. Cardiovascular status did not differ between groups or among tasks except the Older group had a higher MAP immediately after the R9HPT (p = 0.032) compared to the Younger group. Abbreviations: *R9HPT = Right hand 9-hole peg test; L9HPT = Left hand 9-hole peg test; SpO*_*2*_ *= saturation of oxygen; HR = heart rate; MAP = mean arterial pressure.*
(DOCX)

S1 Fig fNIRS optodes placement. The PFC ΔO_2_Hb was obtained using continuous-wave NIRS-system (OctaMon, Artinis, The Netherlands) through application of an eight-channel sparse grid array designed for acquisition over the PFC [1]. The system consists of two receivers (R) and 8 emitters (E) of infrared light at two wavelengths, 730 nm and 850 nm to detect deoxygenated and oxygenated hemoglobin, respectively. The interoptode distance was 30 mm. The light-intensity raw data were sampled at 10 Hz by Oxysoft software (Artinis, The Netherlands) [20,21]. Channels were termed upper (within red band) or lower (within blue band) and medial (inner channels) or dorsolateral (outer channels). One of two sizes of bands were fitted to participants that were either 20 or 25 mm between the medial channels. 1. Paulmurugan K, Vijayaragavan V, Ghosh S, Padmanabhan P, Gulyás B. Brain–computer interfacing using functional near-infrared spectroscopy (fNIRS). Biosensors. 2021 Oct 13;11(10):389.
(DOCX)

S2 Fig Correlations of magnitude of change in O_2_Hb among 8 PFC regions. Spearman correlations analyses were conducted within the Younger and Older group to assess the relationships of ΔO_2_Hb among 8 PFC regions for the R9HPT and the L9HPT. Stronger correlations are shown by a larger size and darker blue colour of circle (see the legend on the right vertical axis of each plot. × indicates non-significance at p > 0.05 and/ indicates non-significance after multiple comparisons were corrected by modified Bonferroni. R9HPT = Right hand 9-hole peg test; L9HPT = Left hand 9-hole peg test; RUpDL = Right Upper Dorsolateral PFC; LUpDL = Left Upper Dorsolateral PFC; RLowDL = Right Lower Dorsolateral PFC; LLowDL = Left lower Dorsolateral PFC; RUpMed = Right Upper Medial PFC; LUpMed = Left Upper Medial PFC; RLowMed = Right Lower Medial PFC; LLowMed = Left Lower Medial PFC.
(DOCX)

S3 Fig Time course of the ΔO_2_Hb during R9HPT and L9HPT in each of the 8 PFC regions for the Younger (S3 Fig A and B) and Older (S3 Fig C and D) groups (shown below). The blue line represents the mean, and the grey vertical lines represent the 95% confidence intervals. The x-axis shows the duration of test in seconds, and the y-axis represents the magnitude of change of O_2_Hb. Overall, increases in the magnitude of ΔO_2_Hb in all 8 regions throughout R9HPT and L9HPT are shown. The downward deflections after 23–24 seconds are likely reflective of fewer adults contributing to these longer time points who had lower neural activity with their corresponding longer durations. Abbreviations: R9HPT = Right hand 9-hole peg test; L9HPT = Left hand 9-hole peg test; RUpDL = Right Upper Dorsolateral PFC; LUpDL = Left Upper Dorsolateral PFC; RLowDL = Right Lower Dorsolateral PFC; LLowDL = Left lower Dorsolateral PFC; RUpMed = Right Upper Medial PFC; LUpMed = Left Upper Medial PFC; RLowMed = Right Lower Medial PFC; LLowMed = Left Lower Medial PFC.
(DOCX)
