# Supplementary material for: Prefrontal cortex connectivity during right and left hand dexterity tests in younger and older adults
Source: PLoS One. 2026 Feb 12;21(2):e0342547. doi: 10.1371/journal.pone.0342547 (PMC12900323; doi:10.1371/journal.pone.0342547)
Supplement: S1 Table — In the Older Group, older age was correlated with a larger ΔO2Hb in 5 out of 8 PFC regions during the L9HPT. Significant values are bolded. Abbreviations: R9HPT: Right hand 9-hole peg test; L9HPT = Left hand 9-hole peg test; PFC = Prefrontal cortex; RUpDL = Right Upper Dorsolateral PFC; LUpDL = Left Upper Dorsolateral PFC; RLowDL = Right Lower Dorsolateral PFC; LLowDL = Left lower Dorsolateral PFC; RUpMed = Right Upper Medial PFC; LUpMed = Left Upper Medial PFC; RLowMed = Right Lower Medial PFC; LLowMed = Left Lower Medial PFC. (DOCX) [file pone.0342547.s001.docx]

**Table S1:** Correlations between age and ΔO_2_Hb in 8 PFC regions.

| PFC region | Younger R9HPT | | Younger L9HPT | | Older R9HPT | | Older L9HPT | |
| --- | --- | --- | --- | --- | --- | --- | --- | --- |
|  | r | p-value | r | p-value | r | p-value | r | p-value |
| RUpDL | .180 | 0.140 | .048 | 0.387 | .362 | 0.140 | **.481** | **0.043** |
| RLowDL | .166 | 0.160 | .062 | 0.356 | .377 | 0.123 | **.602** | **0.008** |
| RUpMed | .119 | 0.239 | .033 | 0.421 | .249 | 0.320 | .362 | 0.139 |
| RLowMed | **.287** | **0.040** | .121 | 0.235 | .346 | 0.160 | **.547** | **0.019** |
| LUpMed | -.172 | 0.151 | -.043 | 0.400 | -.417 | 0.085 | .208 | 0.409 |
| LLowMed | .256 | 0.061 | .158 | 0.172 | .318 | 0.199 | **.567** | **0.014** |
| LUpDL | .148 | 0.187 | -.028 | 0.433 | .113 | 0.654 | .383 | 0.117 |
| LLowDL | .191 | 0.125 | 0.112 | 0.251 | .113 | 0.654 | **.651** | **0.003** |

In the Older Group, older age was correlated with a larger ΔO_2_Hb in 5 out of 8 PFC regions during the L9HPT. Significant values are bolded.

Abbreviations:

*R9HPT: Right hand 9-hole peg test; L9HPT = Left hand 9-hole peg test; PFC = Prefrontal cortex; RUpDL = Right Upper Dorsolateral PFC; LUpDL = Left Upper Dorsolateral PFC; RLowDL = Right Lower Dorsolateral PFC; LLowDL = Left lower Dorsolateral PFC; RUpMed = Right Upper Medial PFC; LUpMed = Left Upper Medial PFC; RLowMed = Right Lower Medial PFC; LLowMed = Left Lower Medial PFC.*
