# Supplementary material for: Prefrontal cortex connectivity during right and left hand dexterity tests in younger and older adults
Source: PLoS One. 2026 Feb 12;21(2):e0342547. doi: 10.1371/journal.pone.0342547 (PMC12900323; doi:10.1371/journal.pone.0342547)
Supplement: S2 Table — Data presented as median and interquartile range. Data presented as median and interquartile range. Cardiovascular status did not differ between groups or among tasks except the Older group had a higher MAP immediately after the R9HPT (p = 0.032) compared to the Younger group. Abbreviations: R9HPT = Right hand 9-hole peg test; L9HPT = Left hand 9-hole peg test; SpO2 = saturation of oxygen; HR = heart rate; MAP = mean arterial pressure. (DOCX) [file pone.0342547.s002.docx]

**Table S2:** Cardiovascular status measured at baseline and after each task. Data presented as median and interquartile range.

| **Physiological outcome** | **Condition** | **Younger** | **Older** | **p value** |
| --- | --- | --- | --- | --- |
| SpO_2_ (%) | Baseline | 99 [98, 100] | 98 [96, 98] | .069 |
|  | Right 9HPT | 99 [98, 100] | 98 [97, 99] | .099 |
|  | Left 9HPT | 98 [98, 99] | 98 [98, 99] | .664 |
| HR (beats/min) | Baseline | 69 [65, 80] | 68 [64, 71] | .650 |
|  | Right 9HPT | 69 [65, 75] | 67 [64, 71] | .335 |
|  | Left 9HPT | 71 [63, 78] | 68 [59, 79] | .539 |
| MAP (mmHg) | Baseline | 90 [82, 97] | 95 [85, 107] | .126 |
|  | Right 9HPT | 87 [81, 93] | 95 [87, 101] | **.032** |
|  | Left 9HPT | 87 [81, 93] | 92 [83, 101] | .222 |

Data presented as median and interquartile range.

Cardiovascular status did not differ between groups or among tasks except the Older group had a higher MAP immediately after the R9HPT (p=0.032) compared to the Younger group.

Abbreviations:

*R9HPT = Right hand 9-hole peg test; L9HPT = Left hand 9-hole peg test; SpO_2_ = saturation of oxygen; HR = heart rate; MAP = mean arterial pressure.*
