# Supplementary material for: Prefrontal cortex connectivity during right and left hand dexterity tests in younger and older adults
Source: PLoS One. 2026 Feb 12;21(2):e0342547. doi: 10.1371/journal.pone.0342547 (PMC12900323; doi:10.1371/journal.pone.0342547)
Supplement: S1 Fig — The PFC ΔO2Hb was obtained using continuous-wave NIRS-system (OctaMon, Artinis, The Netherlands) through application of an eight-channel sparse grid array designed for acquisition over the PFC [1]. The system consists of two receivers (R) and 8 emitters (E) of infrared light at two wavelengths, 730 nm and 850 nm to detect deoxygenated and oxygenated hemoglobin, respectively. The interoptode distance was 30 mm. The light-intensity raw data were sampled at 10 Hz by Oxysoft software (Artinis, The Netherlands) [20,21]. Channels were termed upper (within red band) or lower (within blue band) and medial (inner channels) or dorsolateral (outer channels). One of two sizes of bands were fitted to participants that were either 20 or 25 mm between the medial channels. 1. Paulmurugan K, Vijayaragavan V, Ghosh S, Padmanabhan P, Gulyás B. Brain–computer interfacing using functional near-infrared spectroscopy (fNIRS). Biosensors. 2021 Oct 13;11(10):389. (DOCX) [file pone.0342547.s003.docx]

**Figure S1**: fNIRS optodes placement


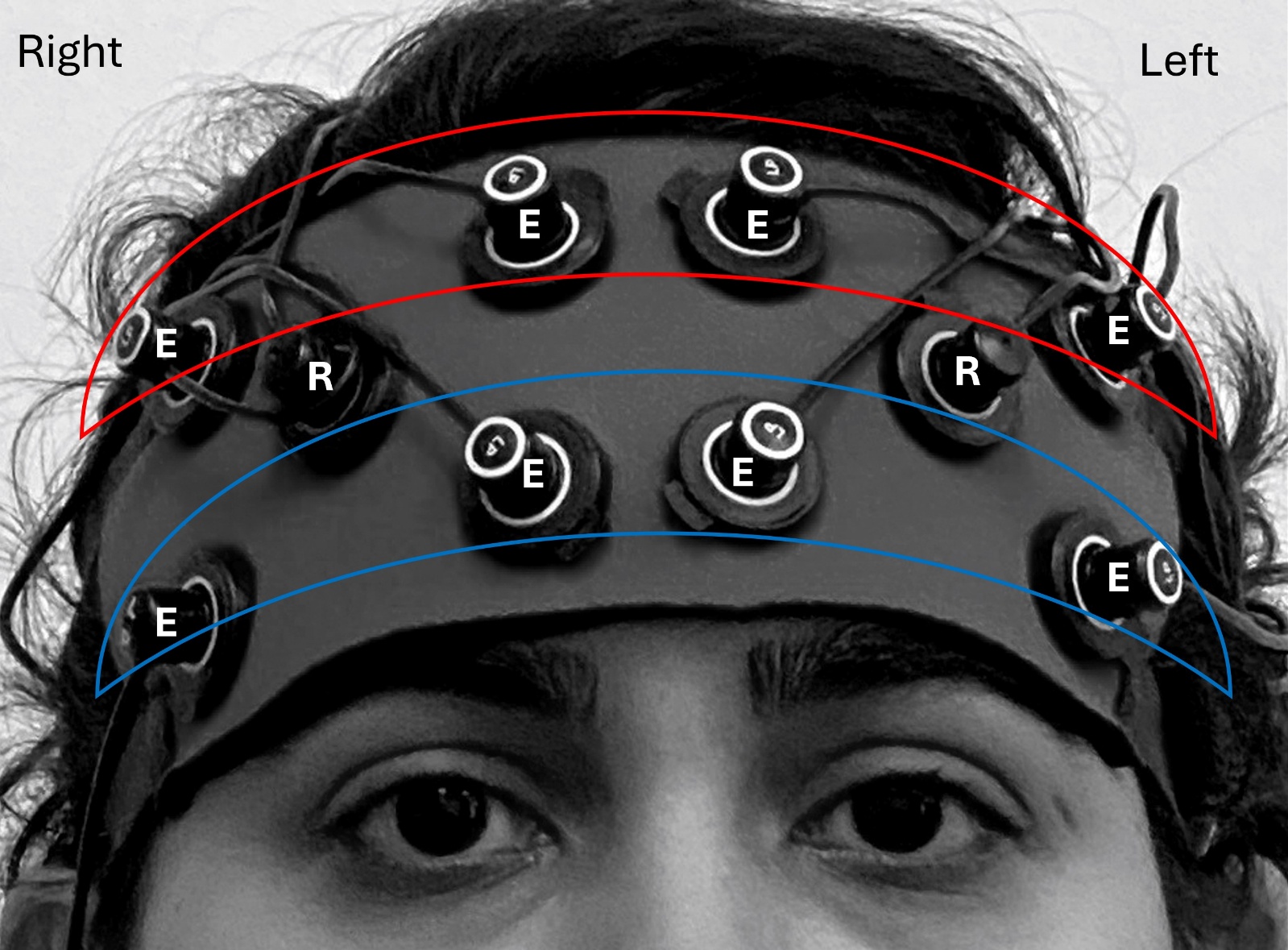


The PFC ΔO_2_Hb was obtained using continuous-wave NIRS-system (OctaMon, Artinis, The Netherlands) through application of an eight-channel sparse grid array designed for acquisition over the PFC [1]. The system consists of two receivers (R) and 8 emitters (E) of infrared light at two wavelengths, 730 nm and 850 nm to detect deoxygenated and oxygenated hemoglobin, respectively. The interoptode distance was 30 mm. The light-intensity raw data were sampled at 10Hz by Oxysoft software (Artinis, The Netherlands) [20,21]. Channels were termed upper (within red band) or lower (within blue band) and medial (inner channels) or dorsolateral (outer channels). One of two sizes of bands were fitted to participants that were either 20 or 25 mm between the medial channels.

1. Paulmurugan K, Vijayaragavan V, Ghosh S, Padmanabhan P, Gulyás B. Brain–computer interfacing using functional near-infrared spectroscopy (fNIRS). Biosensors. 2021 Oct 13;11(10):389
