# Supplementary material for: Prefrontal cortex connectivity during right and left hand dexterity tests in younger and older adults
Source: PLoS One. 2026 Feb 12;21(2):e0342547. doi: 10.1371/journal.pone.0342547 (PMC12900323; doi:10.1371/journal.pone.0342547)
Supplement: S2 Fig — Spearman correlations analyses were conducted within the Younger and Older group to assess the relationships of ΔO2Hb among 8 PFC regions for the R9HPT and the L9HPT. Stronger correlations are shown by a larger size and darker blue colour of circle (see the legend on the right vertical axis of each plot. × indicates non-significance at p > 0.05 and/ indicates non-significance after multiple comparisons were corrected by modified Bonferroni. R9HPT = Right hand 9-hole peg test; L9HPT = Left hand 9-hole peg test; RUpDL = Right Upper Dorsolateral PFC; LUpDL = Left Upper Dorsolateral PFC; RLowDL = Right Lower Dorsolateral PFC; LLowDL = Left lower Dorsolateral PFC; RUpMed = Right Upper Medial PFC; LUpMed = Left Upper Medial PFC; RLowMed = Right Lower Medial PFC; LLowMed = Left Lower Medial PFC. (DOCX) [file pone.0342547.s004.docx]

**Figure S2**: Correlations of magnitude of change in O_2_Hb among 8 PFC regions.

*
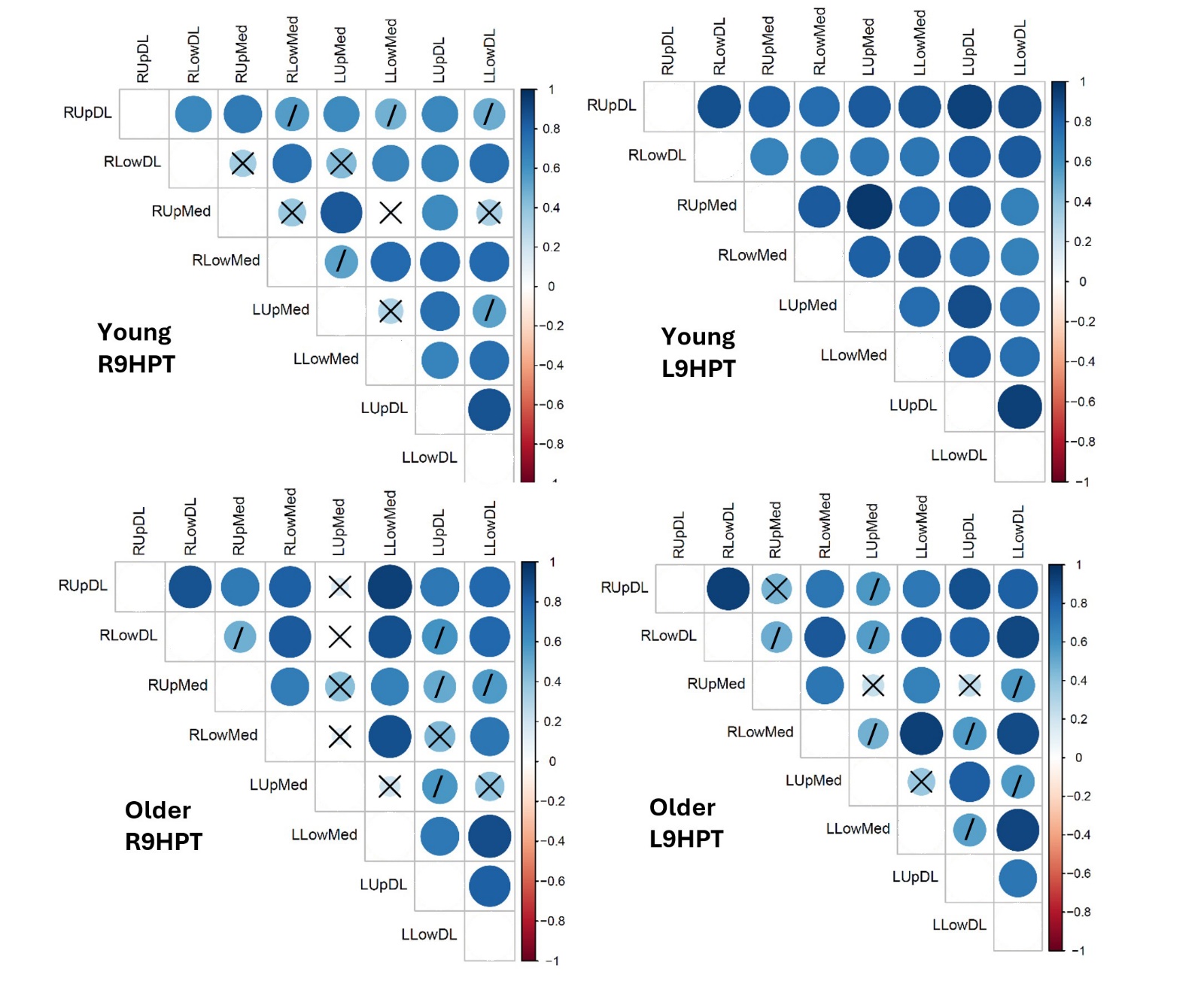
*

Spearman correlations analyses were conducted within the Younger and Older group to assess the relationships of ΔO_2_Hb among 8 PFC regions for the R9HPT and the L9HPT. Stronger correlations are shown by a larger size and darker blue colour of circle (see the legend on the right vertical axis of each plot. × indicates non-significance at p>0.05 and / indicates non-significance after multiple comparisons were corrected by modified Bonferroni.

R9HPT= Right hand 9-hole peg test; L9HPT = Left hand 9-hole peg test; RUpDL = Right Upper Dorsolateral PFC; LUpDL = Left Upper Dorsolateral PFC; RLowDL = Right Lower Dorsolateral PFC; LLowDL = Left lower Dorsolateral PFC; RUpMed = Right Upper Medial PFC; LUpMed = Left Upper Medial PFC; RLowMed = Right Lower Medial PFC; LLowMed = Left Lower Medial PFC.
