# Supplementary material for: Prefrontal cortex connectivity during right and left hand dexterity tests in younger and older adults
Source: PLoS One. 2026 Feb 12;21(2):e0342547. doi: 10.1371/journal.pone.0342547 (PMC12900323; doi:10.1371/journal.pone.0342547)
Supplement: S3 Fig — The blue line represents the mean, and the grey vertical lines represent the 95% confidence intervals. The x-axis shows the duration of test in seconds, and the y-axis represents the magnitude of change of O2Hb. Overall, increases in the magnitude of ΔO2Hb in all 8 regions throughout R9HPT and L9HPT are shown. The downward deflections after 23–24 seconds are likely reflective of fewer adults contributing to these longer time points who had lower neural activity with their corresponding longer durations. Abbreviations: R9HPT = Right hand 9-hole peg test; L9HPT = Left hand 9-hole peg test; RUpDL = Right Upper Dorsolateral PFC; LUpDL = Left Upper Dorsolateral PFC; RLowDL = Right Lower Dorsolateral PFC; LLowDL = Left lower Dorsolateral PFC; RUpMed = Right Upper Medial PFC; LUpMed = Left Upper Medial PFC; RLowMed = Right Lower Medial PFC; LLowMed = Left Lower Medial PFC. (DOCX) [file pone.0342547.s005.docx]

**Figure S3:** Time course of the ΔO_2_Hb during R9HPT and L9HPT in each of the 8 PFC regions for the Younger (**Figure S3 A and B**) and Older (**Figure S3 C and D**) groups (shown below).

The blue line represents the mean, and the grey vertical lines represent the 95% confidence intervals. The x-axis shows the duration of test in seconds, and the y-axis represents the magnitude of change of O_2_Hb. Overall, increases in the magnitude of ΔO_2_Hb in all 8 regions throughout R9HPT and L9HPT are shown. The downward deflections after 23 to 24 seconds are likely reflective of fewer adults contributing to these longer time points who had lower neural activity with their corresponding longer durations.

Abbreviations: R9HPT = Right hand 9-hole test; L9HPT = Left hand 9-hole test; RUpDL = Right Upper Dorsolateral PFC; LUpDL = Left Upper Dorsolateral PFC; RLowDL = Right Lower Dorsolateral PFC; LLowDL = Left lower Dorsolateral PFC; RUpMed = Right Upper Medial PFC; LUpMed = Left Upper Medial PFC; RLowMed = Right Lower Medial PFC; LLowMed = Left Lower Medial PFC.

**Figure S3 A: Younger Adults R9HPT**

Time (sec)

Time (sec)

**Figure S3 B: Younger Adults L9HPT**

Time (sec)

Time (sec)

**Figure S3 C: Older Adults R9HPT**

Time (sec)

Time (sec)

**Figure S3 D: Older Adults L9HPT**

Time (sec)

Time (sec)
